# Mitochondrial and redox abnormalities in autism lymphoblastoid cells: a sibling control study

**DOI:** 10.1096/fj.201601004R

**Published:** 2016-11-18

**Authors:** Shannon Rose, Sirish C. Bennuri, Rebecca Wynne, Stepan Melnyk, S. Jill James, Richard E. Frye

**Affiliations:** * Arkansas Children’s Research Institute, Little Rock, Arkansas, USA; and; † Department of Pediatrics, University of Arkansas for Medical Sciences, Little Rock, Arkansas, USA

**Keywords:** autistic disorder, bioenergetics, glutathione, oxidative stress, UCP2

## Abstract

Autism spectrum disorder (ASD) is associated with physiological abnormalities, including abnormal redox and mitochondrial metabolism. Lymphoblastoid cell lines (LCLs) from some children with ASD exhibit increased oxidative stress, decreased glutathione redox capacity, and highly active mitochondria with increased vulnerability to reactive oxygen species (ROS). Because unaffected siblings (Sibs) of individuals with ASD share some redox abnormalities, we sought to determine whether LCLs from Sibs share ASD-associated mitochondrial abnormalities. We evaluated mitochondrial bioenergetics in 10 sets of LCLs from children with ASD, Sibs, and unrelated/unaffected controls (Cons) after acute increases in ROS. Additionally, intracellular glutathione and uncoupling protein 2 (*UCP2*) gene expressions were quantified. Compared to Sib LCLs, ASD LCLs exhibited significantly higher ATP-linked respiration, higher maximal and reserve respiratory capacity, and greater glycolysis and glycolytic reserve. ASD LCLs exhibited a significantly greater change in these parameters, with acute increases in ROS compared to both Sib and Con LCLs. Compared to Con, both ASD and Sib LCLs exhibited significantly higher proton leak respiration. Consistent with this, intracellular glutathione redox capacity was decreased and *UCP2* gene expression was increased in both ASD and Sib compared to Con LCLs. These data indicate that mitochondrial respiratory function, not abnormal redox homeostasis, distinguishes ASD from unaffected LCLs.—Rose, S., Bennuri, S. C., Wynne, R., Melnyk, S., James, S. J., Frye, R. E. Mitochondrial and redox abnormalities in autism lymphoblastoid cells: a sibling control study.

There is an increased recognition that several physiological abnormalities are related to autism (1), including mitochondrial dysfunction (2, 3). Some studies estimate that 50% or more of children with autism manifest biomarkers of mitochondrial dysfunction (4, 5), while other studies have suggested that 80% of children with autism have abnormal electron transport chain function (6, 7). We recently demonstrated a novel type of mitochondrial dysfunction in approximately one-third of lymphoblastoid cell lines (LCLs) from children with autism spectrum disorder (ASD) compared to LCLs from unaffected controls (Cons). This subgroup of ASD LCLs exhibited increases in respiratory activity, vulnerability to reactive oxygen species (ROS), and uncoupling protein 2 (UCP2) content (8, 9). It is unclear whether mitochondrial dysfunction is driven by other metabolic abnormalities associated with ASD, such as oxidative stress, or whether it is specifically linked to ASD. Unaffected siblings (Sibs) of ASD individuals also demonstrate some redox abnormalities (10). Whether Sibs also exhibit mitochondrial abnormalities is unknown.

To determine the role of redox abnormalities in mitochondrial dysfunction associated with ASD and whether a particular metabolic abnormality is specific for ASD, we compared mitochondrial respiration with and without ROS exposure as well as intracellular glutathione content and *UCP2* gene expression in LCLs from children with ASD, Sibs, and unrelated Cons.

## MATERIALS AND METHODS

### Materials

RPMI 1640 culture medium, penicillin/streptomycin, fetal bovine serum, PBS, and BCA Protein Assay Kit were all obtained from Thermo Fisher Scientific (Waltham, MA, USA). XF DMEM and XF-PS 96-well plates were obtained from Agilent Technologies (Santa Clara, CA, USA). The RNeasy Mini Kit was obtained from Qiagen (Germantown, MD, USA) and the High Capacity cDNA Reverse Transcription Kit and Power SYBR Green PCR Master Mix from Thermo Fisher Scientific. Poly-d-lysine, 2,3-dimethoxy-1,4-napthoquinone (DMNQ), meta-phosphoric acid, and all other chemicals were obtained from Sigma-Aldrich (St. Louis, MO, USA).

### Cell lines and culture

Ten pairs of LCLs derived from multiplex families with 1 male member diagnosed with ASD and 1 unaffected male Sib were obtained from the Autism Genetic Resource Exchange (Los Angeles, CA, USA). Seven unrelated age-matched Con LCLs derived from healthy male donors with no documented behavioral or neurological disorder or first-degree relative with a medical disorder that could involve abnormal mitochondrial function were obtained from Coriell Cell Repository (Camden, NJ, USA). Details of the LCLs are presented in **Table 1**. Seven of the ASD LCLs examined here were previously included as part of a larger cohort that we classified according to mitochondrial phenotype and reported an atypical mitochondrial phenotype in approximately 30% of the ASD LCLs (9). In that study, 3 were characterized as atypical and 4 were characterized as typical, and 3 have not been previously studied. LCLs were maintained in RPMI 1640 culture medium with 15% fetal bovine serum and 1% penicillin/streptomycin in a humidified incubator at 37°C with 5% CO_2_. All ASD LCLs were linked to the results of the reference-standard Autism Diagnostic Observation Schedule (ADOS) assessments of the children from which the LCLs were derived.

**TABLE 1:** Matched LCLs

Unrelated/unaffected Con		ASD		Unaffected Sib	
LCL	Age	LCL	Age	LCL	Age
GM09659	4	AU1393306	3	AU1393305	4
GM11599	9	AU038804	8	AU038803	9
GM15862	11	AU0939303	11	AU0939302	8
GM10153	10	AU1267302	10	AU1267303	7
GM16007	12	AU1348303	12	AU1348302	13
GM09621	8	AU1344302	7	AU1344303	4
GM09621	8	AU1280302	7	AU1280304	1
GM16007	12	AU1215301	12	AU1215305	2
GM11626	13	AU008404	13	AU008405	9
GM11626	13	AU1165302	13	AU1165303	12
Mean ± se	10 ± 0.9		9.6 ± 1.1		6.9 ± 1.4

### Mitochondrial respiration

Mitochondrial oxygen consumption rate and extracellular acidification rate (ECAR) were measured in real time in live intact LCLs using a Seahorse Extracellular Flux (XF) 96 Analyzer (Agilent Technologies) with details previously published (9). Briefly, age- and gender-matched sets of LCLs consisting of ASD, Sib, and Con LCLs were seeded onto poly-d-lysine-coated 96-well XF-PS plates in XF DMEM with at least 3 replicate wells per treatment group. The cells were then treated for 1 h with the redox cycling agent DMNQ, which enters cells and generates both superoxide and hydrogen peroxide similar to levels generated by NADPH oxidase (11). DMNQ (5 mg/ml) was prepared in DMSO and diluted in XF DMEM into 10× stocks and added directly into wells of the XF plate incubated for 1 h at 37°C in a non-CO_2_ incubator with final concentrations of 5, 10, and 15 μM DMNQ. Previously, we demonstrated that these concentrations of DMNQ increase oxidative stress in LCLs (9).

### Quantification of intracellular glutathione

Intracellular free reduced glutathione (GSH) and oxidized glutathione (GSSG) were quantified in each of the 27 cell lines by HPLC as previously described (12). Briefly, approximately 5 million untreated cells from each cell line were pelleted and snap-frozen on dry ice and stored at −80°C. Upon thawing, cells were lysed by sonication in ice-cold PBS, followed by the addition of ice-cold 10% meta-phosphoric acid. After a 30-min incubation on ice, samples were centrifuged for 15 min at 18,000 *g* at 4°C. Results are expressed per protein using the BCA Protein Assay Kit.

### *UCP2* gene expression

Total RNA was isolated from 5 million untreated cells from each of the 27 cell lines using the RNeasy Mini Kit following the manufacturer’s protocol. cDNA synthesis (2 µg per 20 µl reaction mix) was performed using the High Capacity cDNA Reverse Transcription Kit as indicated by the manufacturer. Quantitative PCR reactions were performed using Power SYBR Green PCR Master Mix on an ABI 7900HT Fast Real-Time PCR system (Thermo Fisher Scientific). Relative quantification was performed to the housekeeping gene hypoxanthine phosphoribosyltransferase 1 (*HPRT1*). Primers for *UCP2* (F: 5′–TCCTGAAAGCCAACCTCATG–3′; R: 5′–GGCAGAGTTCATGTATCTCGTC–3′) and *HPRT1* (F: 5′–TGCTGAGGATTTGGAAAGGG–3′; R: 5′–ACAGAGGGCTACAATGTGATG–3′) were designed using the real-time PCR tool from IDT DNA (*http://www.idtdna.com/*).

### Statistical analyses

Respiratory parameters were analyzed using mixed-model regression (SAS 9.3; SAS Institute, Cary, NC, USA), similar to our previous studies (8, 9). Respiratory parameters from ASD LCLs were compared to paired Sib and Con LCLs with group as the between-group effect and DMNQ as the within-group repeated factor. DMNQ was modeled to the second power to account for the curvilinear relationship. Presented are the overall differences between the groups (group effect), the linear and quadratic effects of DMNQ, and interaction between the group and the linear and quadratic effect of DMNQ. For all models, random effects included the intercept and DMNQ. Significance was evaluated with *F* tests. Planned *post hoc* orthogonal contrasts were used when the interaction was significant. We also investigated whether ASD symptoms were linked to changes in oxygen consumption rate and ECAR parameters only in the ASD LCLs. In these analyses, we examined the ADOS scores of Total Communication and Social Symptoms and Total Stereotyped Behaviors and Restricted Interests (SBRI) separately. The change in overall parameters as well as the interaction with the linear effect of DMNQ were investigated. Differences between glutathione content and *UCP2* expression across groups were performed similar to the analyses previously described.

## RESULTS

### Oxygen consumption

**Figure 1.**
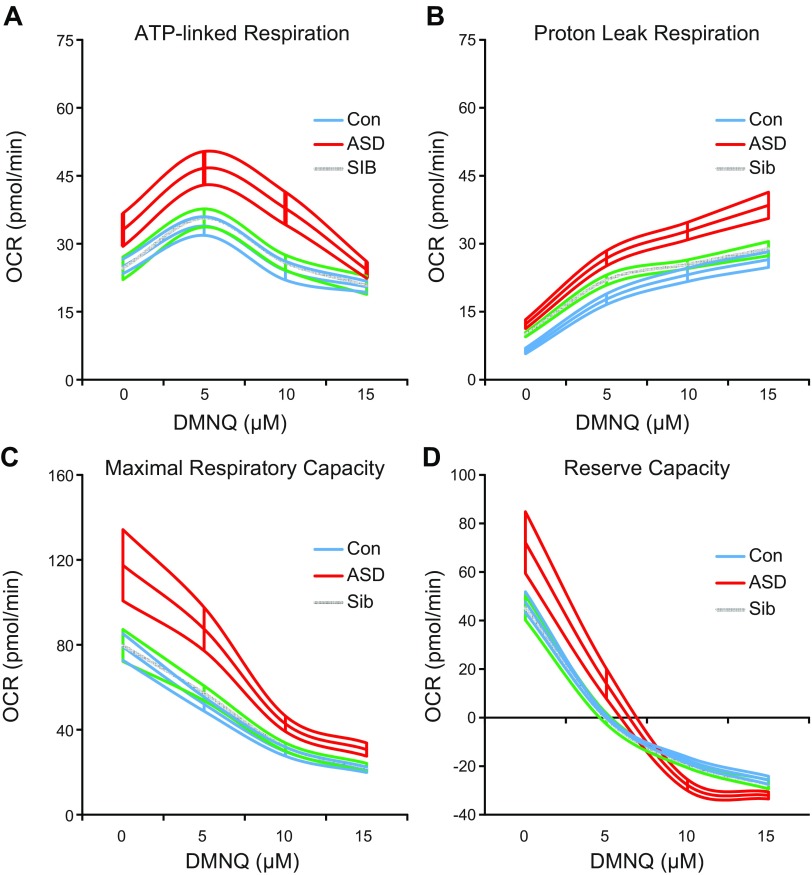
Mitochondrial respiratory parameters. Mean curves of mitochondrial parameters with 1 h exposure to DMNQ are outlined by upper and lower standard errors. *A*) Both overall [*F*(2,423) = 7.60, *P* < 0.001] and change in ATP-linked respiration [*F*(2,423) = 3.64, *P* < 0.05] differed across groups, with ASD LCLs exhibiting markedly higher overall ATP-linked respiration compared to Sib [*t*(423) = 3.61, *P* < 0.001] and Con [*t*(423) = 3.03, *P* < 0.005] LCLs and greater change in ATP-linked respiration with DMNQ compared to Sib [*t*(423) = 2.03, *P* < 0.05] and Con [*t*(405) = 2.55, *P* = 0.01] LCLs. *B*) Overall proton leak [*F*(2,423) = 12.07, *P* < 0.0001] differed across groups, with lower overall proton leak in Con compared to ASD [*t*(423) = 4.86, *P* < 0.0001] and Sib [*t*(423) = −3.19, *P* < 0.005] LCLs. *C*) Overall maximal respiratory capacity differed across groups [*F*(2,423) = 25.34, *P* < 0.0001], with higher maximal respiratory capacity in ASD compared to Sib [*t*(423) = 6.26, *P* < 0.001] and Con [*t*(423) = 5.97, *P* < 0.001] LCLs. *D*) Both overall [*F*(2,423) = 20.77, *P* < 0.001] and change in reserve capacity with DMNQ [*F*(2,423) = 5.67, *P* < 0.005] differed across groups. Reserve capacity was markedly higher in ASD compared to Sib [*t*(423) = 6.01, *P* < 0.0001] and Con [*t*(423) = 4.93, *P* < 0.001] LCLs, and decrease in reserve capacity with DMNQ was greater in ASD compared to Sib [*t*(423) = 3.10, *P* < 0.005] and Con [*t*(405) = 2.67, *P* < 0.01] LCLs.

Overall ATP-linked respiration, maximal respiratory capacity, and reserve capacity were significantly greater in ASD compared to both Sib and Con LCLs (*P* < 0.005 for all). At baseline (0 DMNQ), ATP-linked respiration was 35 and 32% higher in ASD LCLs compared to Sib and Con LCLs, respectively (**Fig. 1*A***). While ASD and Sib LCLs are discordant for abnormalities in mitochondrial respiratory parameters linked to ATP production, they exhibited similar proton leak respiration (Fig. 1*B*). Maximal respiratory capacity at baseline was approximately 48% higher in ASD than both Sib and Con LCLs (Fig. 1*C*). Mitochondrial reserve capacity at baseline was 60% higher in ASD than Sib and 52% higher than Con LCLs (Fig. 1*D*). The increase in ATP-linked respiration and the decrease in reserve capacity with increased ROS were greater in ASD than in Sib and Con LCLs (*P* < 0.05 for all). Compared to Con LCLs, baseline proton leak respiration was elevated 93% in ASD and 57% in Sib LCLs, and proton leak respiration was significantly higher overall in ASD and Sib compared to Con LCLs (*P* < 0.005 for all).

### Glycolysis

**Figure 2.**
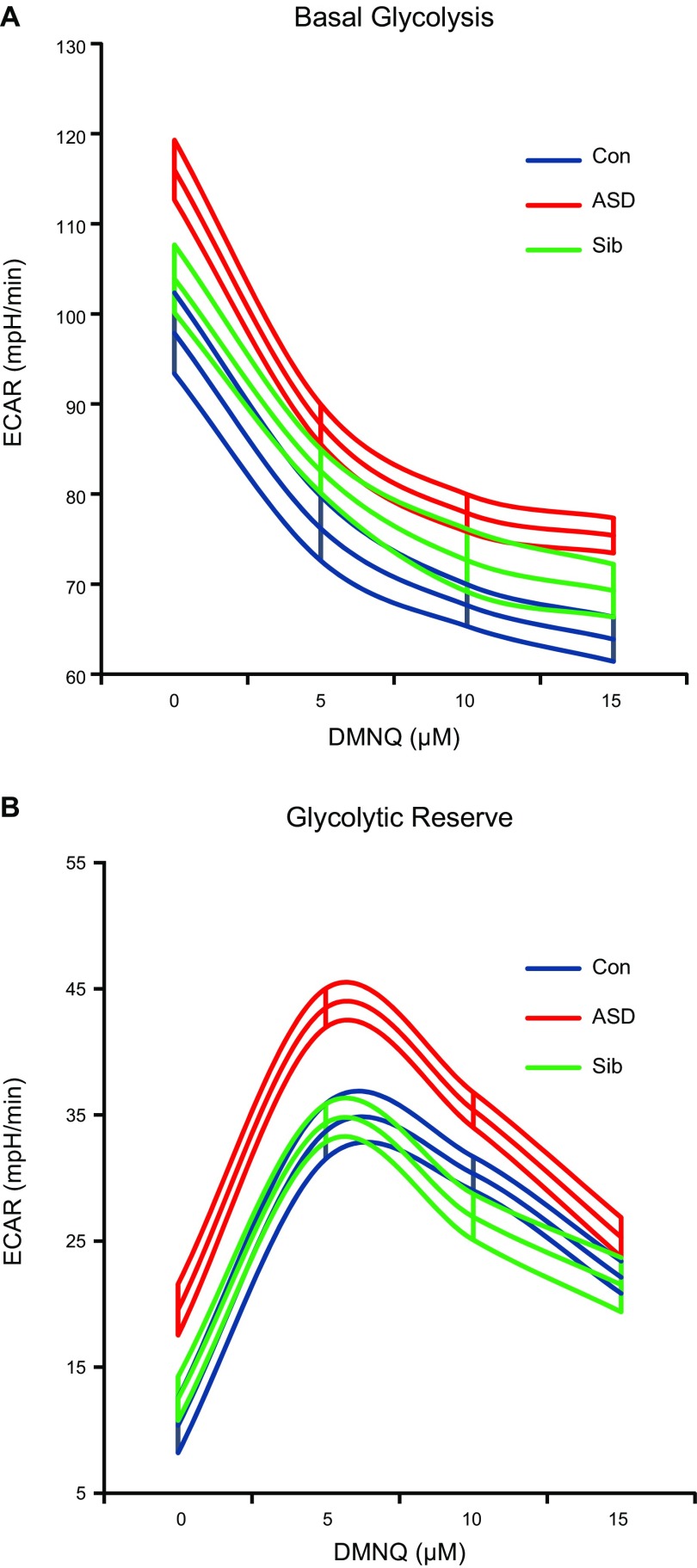
Glycolytic parameters. Mean curves of glycolytic parameters with 1 h exposure to DMNQ are outlined by upper and lower standard errors. *A*) Both overall [*F*(2,370) = 5.34, *P* < 0.01] and change in glycolysis [*F*(2,370) = 5.77, *P* < 0.005] differed across groups. Overall glycolysis was markedly higher in ASD compared to Sib [*t*(370) = 2.57, *P* = 0.01] and Con [*t*(370) = 3.03, *P* < 0.005] LCLs, and decrease in glycolysis with DMNQ was significantly greater in ASD compared to Sib [*t*(370) = 2.96, *P* < 0.005] and Con [*t*(370) = 2.95, *P* = 0.005] LCLs. *B*) LCL groups differed in overall glycolytic reserve [*F*(2,370) = 4.15, *P* < 0.05]. Glycolytic reserve was markedly higher in ASD compared to Sib [*t*(370) = 2.28, *P* < 0.05] and Con [*t*(370) = 2.66, *P* < 0.05] LCLs.

Glycolytic parameters were assessed during the same assay using the ECAR and are presented in **Fig. 2**. Overall, both basal glycolysis and glycolytic reserve were significantly elevated in ASD compared to Sib and Con LCLs (*P* < 0.05 for all). At baseline (0 DMNQ), basal glycolysis was 19 and 12% higher in ASD compared to Sib and Con LCLs, respectively (Fig. 2*A*). Glycolytic reserve at baseline was approximately 89 and 56% higher in ASD than Sib and Con LCLs, respectively (Fig. 2*B*). The decrease in basal glycolysis with DMNQ was greater in ASD than in Sib and Con LCLs (*P* ≤ 0.005 for all).

### Correlations with SBRI scores

**Figure 3.**
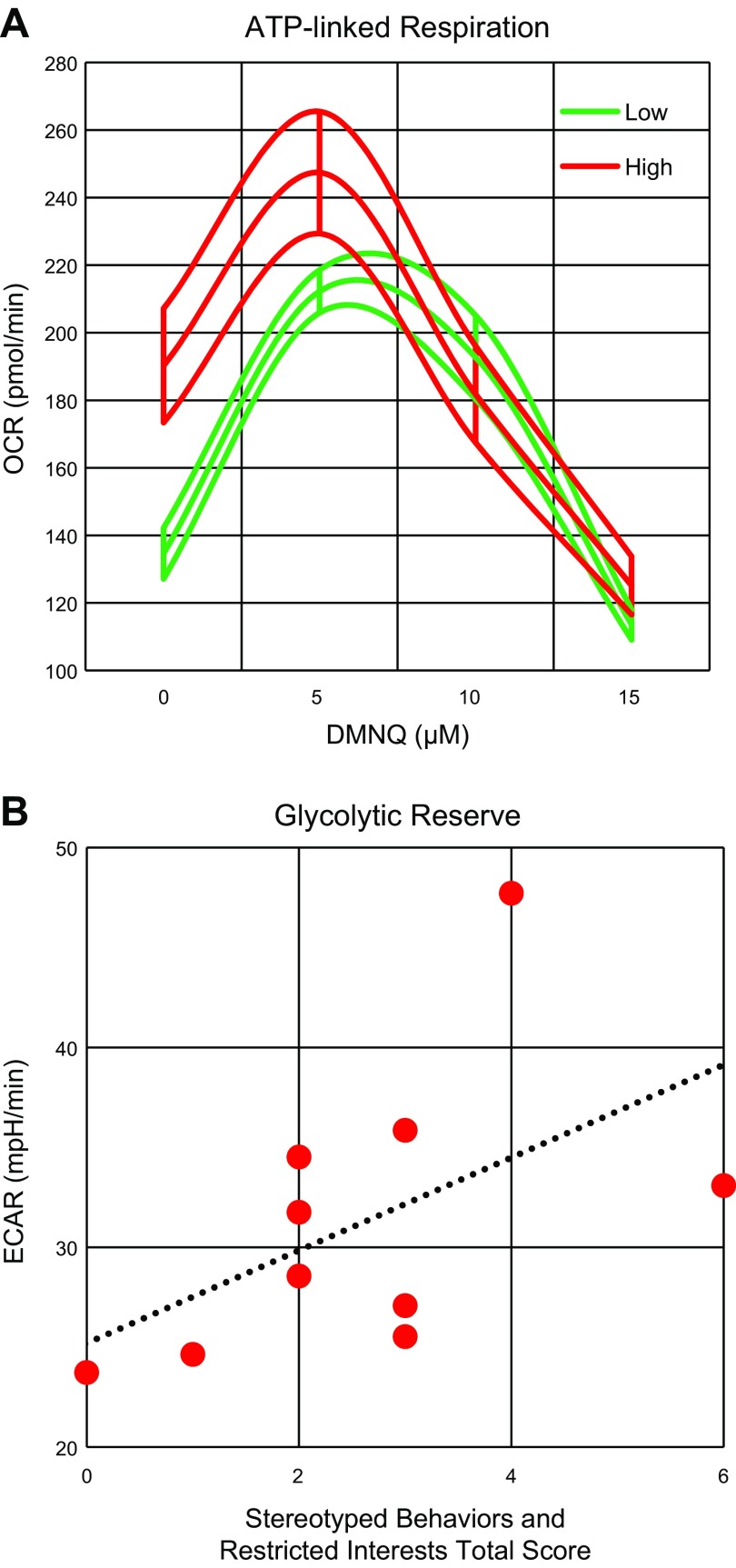
SBRI total scores correlate with bioenergetics. *A*) Effect of DMNQ on ATP-linked respiration was dependent on SBRI total score [*F*(1,131) = 6.60,*P* = 0.01]. Mean curves of ATP-linked respiration with 1 h exposure to DMNQ are outlined by upper and lower standard errors. Those with more severe stereotyped and repetitive behaviors (red lines) started out with higher ATP-linked respiration and had greater drop in ATP-linked respiration than individuals with relatively more mild repetitive behaviors (green lines). *B*) SBRI total score was related to overall glycolytic reserve [*F*(1,125) = 5.60,*P* < 0.05] such that more severe scores were related to higher glycolytic reserve.

The effect of DMNQ on ATP-Linked respiration was dependent on SBRI score. **Figure 3*A*** demonstrates that those with higher SBRI scores (*i.e.,* more behaviors; red lines) started out with a higher ATP-linked respiration and had a more significant drop in ATP-linked respiration than individuals with relatively more mild repetitive behaviors (green lines). The SBRI score was also significantly associated with overall glycolytic reserve (*P* < 0.05) such that more severe scores were related to a higher glycolytic reserve (Fig. 3*B*). No other associations between ADOS scores and any other end points were found.

### Intracellular glutathione

**Figure 4.**
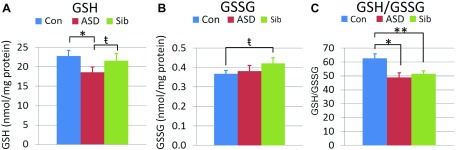
Intracellular glutathione parameters. *A*) GSH differed across groups [*F*(2,18) = 8.95, *P* < 0.01] as ASD LCLs exhibited lower GSH compared to Sib [*t*(18) = 2.46, *P* < 0.05] and Con [*t*(18) = 4.21, *P* < 0.001] LCLs. *B*) GSSG differed across groups [*F*(2,18) = 2.27, *P* = 0.05] as Sib LCLs exhibited greater GSSG than Con [*t*(18) = 2.45, *P* < 0.05] LCLs. *C*) GSH/GSSG differed across groups [*F*(2,18) = 10.57, *P* < 0.001] due to lower GSH/GSSG in ASD [*t*(18) = 4.27, *P* < 0.001] and Sib [*t*(18) = 3.61, *P* < 0.01] LCLs compared to Con LCLs. *P < 0.001, **P < 0.01; ^ŧ^*P* < 0.05.

The glutathione thiol/disulfide redox buffer (GSH/GSSG) is the major mechanism responsible for maintaining a reduced intracellular microenvironment. Intracellular free GSH and GSSG were quantified by HPLC. ASD LCLs exhibited 14% less intracellular GSH compared to Sib (*P* < 0.05) and 19% less GSH compared to Con LCLs (*P* < 0.001; **Fig. 4*A***). Oxidized GSSG was 15% higher in Sib compared to Con LCLs (*P* < 0.005; Fig. 4*B*). The glutathione redox ratio (GSH/GSSG) was decreased by 22% in ASD (*P* < 0.001) and 18% in Sib (*P* < 0.01) compared to Con LCLs (Fig. 4*C*).

### Gene expression

**Figure 5.**
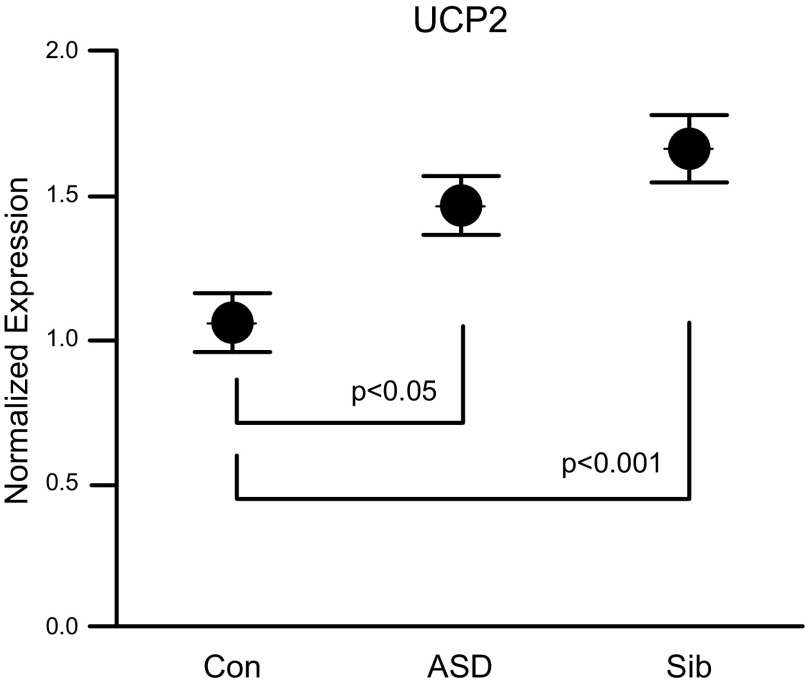
*UCP2* gene expression normalized to housekeeping gene *HPRT1.* *UCP2* expression was significantly different across groups [*F*(2,36) = 7.04, *P* < 0.005], with ASD [*t*(36) = 2.51, *P* < 0.05] and Sib LCLs [*t*(36) = 3.74, *P* < 0.001] demonstrating significantly higher *UCP2* expression compared to Con LCLs.

*UCP2,* a major regulatory mechanism responsible for reducing ROS at the inner mitochondrial membrane, is known to be up-regulated in response to mitochondrial oxidative stress to relieve the proton gradient across the inner mitochondrial membrane (13–15). *UCP2* gene expression was examined by quantitative PCR and was found to be elevated by 38% in ASD (*P* < 0.05) and 57% in Sib LCLs (*P* < 0.001) compared to Con LCLs (**Fig. 5**).

## DISCUSSION

To our knowledge, this report is the first to compare mitochondrial respiration between LCLs from children with ASD and their Sibs. We report that mitochondrial parameters related to ATP production (ATP-linked respiration and maximal respiratory and reserve capacity) differ significantly between ASD and Sib LCLs, with ASD LCLs exhibiting significantly greater mitochondrial respiration compared to Sib LCLs. Furthermore, ASD mitochondria are more sensitive to an acute increase in ROS than Sibs, exhibiting a greater loss in reserve capacity upon DMNQ exposure. These data suggest that mitochondrial overactivity and hypersensitivity to acute oxidative stress is specifically associated with ASD and support several reports in the literature of mitochondrial overactivity in some children with autism, including greater-than-normal activity of complex I in muscle (16) and complex IV in muscle (17, 18), skin, (5) and brain (19). ASD LCLs also exhibited greater glycolysis and glycolytic capacity compared to Sibs. Glycolysis may be up-regulated in the ASD group as a compensatory mechanism to supply pyruvate to the hyperactive electron transport chain or to simply fill an increased ATP demand.

We examined the role of oxidative stress and redox abnormalities in mitochondrial overactivity by examining intracellular glutathione content and *UCP2* expression in LCLs. Both Sib and ASD LCLs exhibit increased proton leak respiration, decreased glutathione-mediated redox potential (GSH/GSSG), and up-regulated *UCP2* expression, which indicates atypical redox metabolism and is consistent with a more oxidized intracellular microenvironment in both ASD and Sib LCLs and with our earlier findings of redox abnormalities in plasma (10). Thus, it is not abnormal redox homeostasis *per se* that distinguishes ASD from Con LCLs but rather the overall respiratory rate and how ASD LCL mitochondria respond to oxidative stress.

Mitochondrial dynamics including fusion, fission, replication, and mitophagy, and mitochondrial activity (Krebs cycle and oxidative phosphorylation) are regulated by multiple nutrient and stress-sensing signaling pathways so that ATP production by the mitochondria remains balanced with cellular energy demands and fuel availability (20–22). Indeed, mitochondria number, size, and even composition of the electron transport chain components vary in different cell types and tissues in accordance with tissue energy demands (23, 24). Chronic oxidative stress in the ASD LCLs likely adds to the energy demand and over time results in adaptive changes in mitochondrial dynamics/composition to meet the ATP demand. In ASD LCLs with hyperactive mitochondria, the increased homeostatic set point of mitochondrial ATP production results in a decreased ability to respond to further energy demands. The significant up-regulation of *UCP2* expression in Sib LCLs compared to Cons, although not significantly different than ASD, suggests that Sib LCLs may respond more efficiently than ASD LCLs to increased oxidative stress brought on by environmental stressors by compensating for increased mitochondrial ROS production that would likely occur during an environmental insult.

It is of significant interest to associate these findings with severity of core and associated symptoms of ASD. Interestingly, we found a significant association between ADOS SBRI scores and the ATP-linked respiratory response to DMNQ such that higher SBRI was related to higher ATP-linked respiration at baseline and a greater drop in ATP-linked respiration with DMNQ. SBRI was also associated with glycolytic reserve such that more severe SBRI scores were related to higher glycolytic reserve. Because this study is limited by a small sample size, these preliminary findings should be confirmed in a larger cohort of samples, as well as in isolated peripheral blood mononuclear cells from ASD–Sib pairs. The results of this study are consistent with the notion of a novel type of mitochondrial dysfunction characterized by increased respiration and perturbation of redox-sensitive bioenergetic regulatory pathways is specific for ASD and may possibly be related to etiological processes.

## Abbreviations

ADOS: Autism Diagnostic Observation Schedule
ASD: autism spectrum disorder
Con: control
DMNQ: 2,3-dimethoxy-1,4-napthoquinone
ECAR: extracellular acidification rate
GSH: reduced glutathione
GSSG: oxidized glutathione
*HPRT1*: hypoxanthine phosphoribosyltransferase 1
LCL: lymphoblastoid cell line
ROS: reactive oxygen species
SBRI: Stereotyped Behaviors and Restricted Interests
Sib: sibling
UCP2: uncoupling protein 2
